# The Role of *MbEGS1* and *MbEGS2* in Methyleugenol Biosynthesis by *Melaleuca bracteata*

**DOI:** 10.3390/plants12051026

**Published:** 2023-02-24

**Authors:** Yongsheng Lin, Ziwen Qiu, Xiaojie Lin, Yingxiang Wu, Xianqian Niu, Guanwen Yin, Dandan Shao, Xuwen Xiang, Yongyu Li, Chao Yang

**Affiliations:** 1 College of Horticulture, Fujian Agriculture and Forestry University, Fuzhou 350002, China; 2 Institute of Natural Products of Horticultural Plants, Fujian Agriculture and Forestry University, Fuzhou 350002, China; 3 Qingyuan Agricultural Science and Technology Extension Service Center, Qingyuan 511518, China; 4 Fujian Institute of Tropical Crops, Zhangzhou 363001, China

**Keywords:** *Melaleuca bracteata*, methyleugenol, eugenol synthase, biosynthesis, transient overexpression, VIGS

## Abstract

Many aromatic plant volatile compounds contain methyleugenol, which is an attractant for insect pollination and has antibacterial, antioxidant, and other properties. The essential oil of *Melaleuca bracteata* leaves contains 90.46% methyleugenol, which is an ideal material for studying the biosynthetic pathway of methyleugenol. Eugenol synthase (EGS) is one of the key enzymes involved in the synthesis of methyleugenol. We recently reported two eugenol synthase genes (*MbEGS1* and *MbEGS2*) present in *M. bracteata*, where *MbEGS1* and *MbEGS2* were mainly expressed in flowers, followed by leaves, and had the lowest expression levels in stems. In this study, the functions of *MbEGS1* and *MbEGS2* in the biosynthesis of methyleugenol were investigated using transient gene expression technology and virus-induced gene silencing (VIGS) technology in *M. bracteata*. Here, in the *MbEGSs* genes overexpression group, the transcription levels of the *MbEGS1* gene and *MbEGS2* gene were increased 13.46 times and 12.47 times, respectively, while the methyleugenol levels increased 18.68% and 16.48%. We further verified the function of the *MbEGSs* genes by using VIGS, as the transcript levels of the *MbEGS1* and *MbEGS2* genes were downregulated by 79.48% and 90.35%, respectively, and the methyleugenol content in *M. bracteata* decreased by 28.04% and 19.45%, respectively. The results indicated that the *MbEGS1* and *MbEGS2* genes were involved in the biosynthesis of methyleugenol, and the transcript levels of the *MbEGS1* and *MbEGS2* genes correlated with the methyleugenol content in *M. bracteata*.

## 1. Introduction

Phenylpropenes, such as eugenol, isoeugenol, chavicol, and their derivatives, are secondary metabolites in a wide range of plants [1,2]. Some phenylpropene compounds are toxic to animals and microorganisms, and a number of plants synthesize phenylpropenes in their organs to attract insects for pollination or to protect them from herbivores and microorganisms [3,4]. Interestingly, *Clarkia breweri* emits a volatile mixture containing eugenol, isoeugenol, and their methyl derivatives [5]. *Ocimum basilicum* synthesizes and releases high levels of eugenol in the glands on the leaves and flower surfaces [1,6]. The flowers of *Petunia hybrida* emit a volatile mixture containing high levels of isoeugenol and low levels of eugenol [7]. Among them, eugenol has been demonstrated to act as an herbivory inhibitor, nematicide, and fungicide [8,9,10], while methyleugenol is a significant insect pollinator attractant for many flowers, particularly for pollinating moths and beetles, as well as a female pheromone mimic for several kinds of fruit flies [11]. Additionally, phenylpropene has a pleasant aroma and flavor, and humans have used plant materials containing phenylpropene as flavorings, preservatives, perfumes, and for the extraction of plant essential oils [3]. Consequently, it is very important to study the mechanisms that regulate the biosynthesis of phenylpropene compounds in different plants to gain a better understanding of how phenylpropene compounds are synthesized as well as the secondary metabolism of plants.

**Figure 1:**
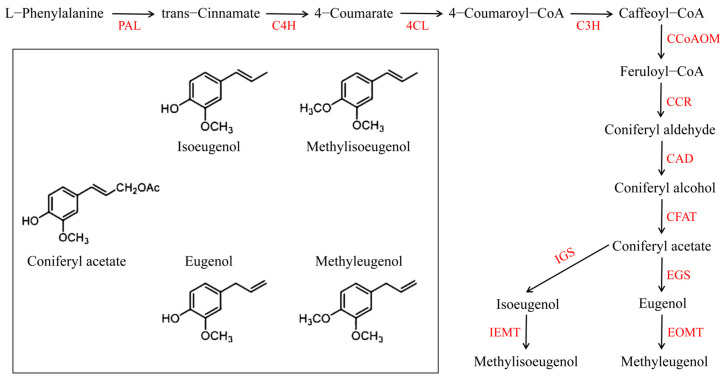
The biosynthesis pathways of methyleugenol.

The biosynthesis pathways of phenylpropenes have been clearly defined; they are derived from the biosynthesis pathways of phenylpropanoids/benzenoids and are the second largest aromatic compounds found in plants [12,13]. The biosynthesis originates from the shikimic acid pathway [14]. In the biosynthesis pathway of phenylpropenes, phenylalanine ammonia-lyase (PAL) uses phenylalanine as substrate to form trans-cinnamic acid. Next, the 4-hydroxycinnamic acid (C4H) converts trans-cinnamic acid to 4-coumarate [15,16,17,18] (Figure 1). It is the key step in the biosynthesis of the phenylpropanoids/benzenoids before entering different metabolic pathways. Subsequently, different metabolic pathways are further modified under the catalysis of various enzymes to form intermediates, such as lignin, defensive substances, and pigments [19,20]. Among them, some intermediates undergo hydroxylation, methylation, and acylation to produce volatile compounds such as aldehydes, alcohols, alkanes, alkenes, ethers, and esters [21,22].

*Melaleuca bracteata* is an evergreen tree belonging to the *Melaleuca* genus of Myrtaceae. It is an aromatic tree species with high ornamental value, commonly known as the black tea-tree/river tea-tree or mock olive [23]. *M. bracteata* leaves are rich in essential oils and have antioxidant, antibacterial, antimicrobial, anticancer, and anti-inflammatory effects [24,25,26]. According to anti-quorum sensing evaluation, the main bioactive component of *M. bracteata* essential oil has been isolated and identified as methyleugenol, and the relative content can be as high as 90.46% [27,28]. Therefore, *M. bracteata* is a good material for studying the biosynthetic pathway of methyleugenol. The biosynthesis of methyleugenol is one of the branches of biosynthesis in the phenylpropanoids/benzenoids pathway. This branch uses coniferyl alcohol as the starting substrate (Figure 1). Coniferyl alcohol acyltransferase (CFAT) uses coniferyl alcohol as substrate to form coniferyl acetate. Next, eugenol synthetase (EGS) converts coniferyl alcohol to eugenol. Finally, eugenol-O-methyltransferase (EOMT) converts eugenol to methyleugenol. It is only through the conversion of coniferyl alcohol into enough eugenol by EGS that methyleugenol can be effectively synthesized. According to previous studies, despite a large amount of methyleugenol being detected in the essential oil of *M. bracteata* leaves, eugenol, isoeugenol, and methylisoeugenol were rarely detected [27,29,30,31]. Therefore, elucidating the mechanism of *EGS* genes in the metabolic pathway of methyleugenol in *M. bracteata* is of great importance for understanding the efficient synthesis of methyleugenol in *M. bracteata*.

EGS is a member of the PIP gene family and is NADPH dependent and has reductase activity. The PIP gene family is named after three members, pinoresinollariciresinol reductase (PLR), isoflavone reductase (IFR), and phenylcoumaran benzylic ether reductase (PCBER), which were discovered originally. The PIP gene family plays important roles in secondary metabolism, growth regulation, and the response to environmental stress in plants [18,32,33,34]. In our previous study, we successfully cloned two eugenol synthase genes (*MbEGS1* and *MbEGS2*) from *M. bracteate*. We found that *MbEGS1* and *MbEGS2* are duplicate genes, encoding 320 and 323 amino acids, respectively. MbEGS1 and MbEGS2 share 72.43% amino acid sequence similarity. Meanwhile, we also performed heterologous transient overexpression experiments on *Fragaria vesca* fruits. The results showed that overexpression of *MbEGS1* and *MbEGS2* in *Fragaria vesca* fruits could positively regulate the synthesis of eugenol [35]. To further explore whether the efficient generation of methyleugenol in *M. bracteata* is related to *MbEGS1* and *MbEGS2*, we used a homologous transient overexpression technique and a virus-induced gene silencing technique (VIGS) to analyze the overexpression and silencing of the *MbEGS1* and *MbEGS*2 genes in *M. bracteata*. We used GC/MS and qRT-PCR techniques to analyze the changes in eugenol and methyleugenol content and genes expression level in the leaves of *M. bracteata* to investigate the roles of *MbEGS1* and *MbEGS2* in the methyleugenol biosynthesis pathway in plants.

## 2. Results

### 2.1. Verification of Recombinant M. bracteata

**Figure 2:**
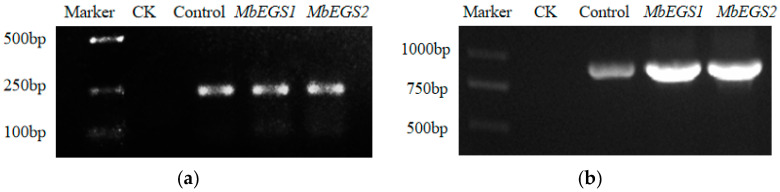
PCR results for the *GUS* reporter gene (**a**) and TRV virus detection (**b**).

Using the untreated *M. bracteata* leaf cDNA as the control template (CK), the *GUS* gene in the overexpression treatment groups and the TRV virus in the VIGS treatment groups were detected by PCR. The results were detected by a 1.0% agarose gel. The results clearly showed that the *GUS* reporter gene and TRV virus were not detected in the control group (CK), whereas *GUS* was detected in the pNC-Cam1304-MCS35S-Control, pNC-Cam1304-MCS35S-*MbEGS1,* and pNC-Cam1304-MCS35S-*MbEGS2* groups (Figure 2a), and the TRV virus was detected in the pNC-TRV2-Control, pNC-TRV2-*MbEGS1,* and pNC-TRV2-*MbEGS2* groups (Figure 2b). Based on these results, it is clear that recombinant pNC-Cam1304-MCS35S-*EGSs* and viral vector pTRV2-*EGSs* can be efficiently replicated and transmitted in *M. bracteata* plants.

### 2.2. Overexpression of MbEGSs in M. bracteata

At 48 h after infection, the transcription level of *MbEGSs* was measured using qRT-PCR in leaves inoculated with pNC-Cam1304-MCS35S-*MbEGSs* to determine whether *MbEGSs* were overexpressed. Additionally, GC-MS was used to detect whether changes in the transcription level of *MbEGSs* genes affected the contents of eugenol and methyleugenol in leaves.

**Figure 3:**
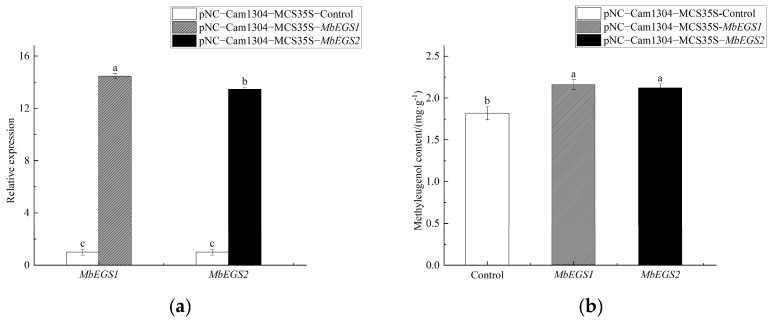
*MbEGSs* genes transcription level analysis (**a**) and contents of methyleugenol (**b**) in the overexpression treatment groups. Note: Different lowercase letters indicate significant differences (*p* < 0.05).

The qRT-PCR results showed that the relative expression levels of the *MbEGS1* and *MbEGS2* genes in leaves increased significantly after transient transformation of the *MbEGS1* and *MbEGS2* overexpression vectors compared to the pNC-Cam1304-MCS35S-Control group. The transcription level of *MbEGS1* was increased 13.46 times in the pNC-Cam1304-MCS35S-*MbEGS1* group. In the pNC-Cam1304-MCS35S-*MbEGS2* group, the transcription level of *MbEGS2* was increased 12.47 times (Figure 3a). The GC-MS results showed that as the transcription levels of the *MbEGS1* and *MbEGS2* genes increased, and the methyleugenol content in leaves significantly increased by 18.68% and 16.48%, respectively (Figure 3b). After comparing the GC-MS total ion chromatograms of the overexpression treatment groups with the NIST 2011 mass spectrometry database, we found that the matching degree for eugenol was all below 700. Therefore, it cannot be accurately identified and the eugenol content cannot be calculated.

### 2.3. VIGS of MbEGSs in M. bracteata

**Figure 4:**
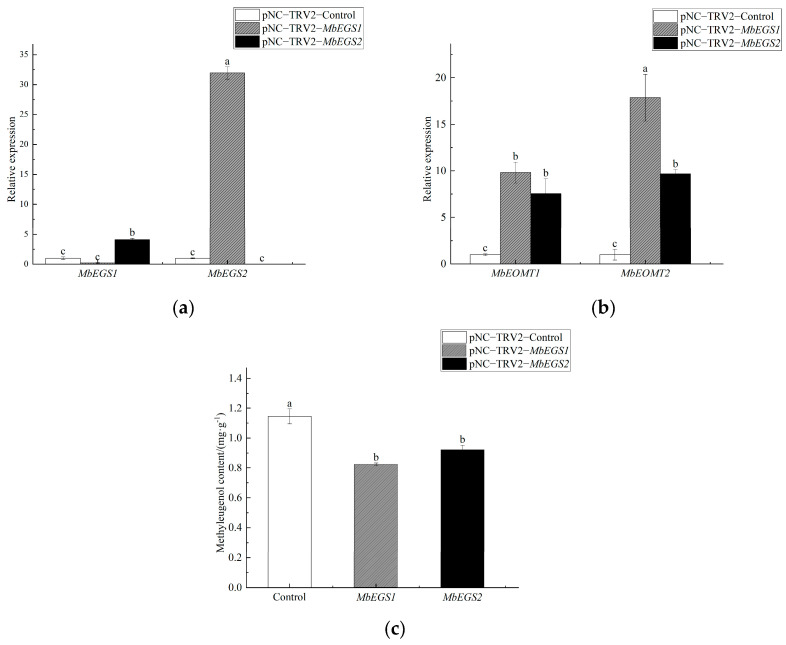
Transcription level analysis of *MbEGSs* genes (**a**) and *MbEOMTs* genes (**b**), and contents of methyleugenol (**c**) in the VIGS treatment groups. Note: Different lowercase letters indicate significant differences (*p* < 0.05).

To further clarify the role of *MbEGSs* genes in methyleugenol biosynthesis by *M. bracteata,* we used VIGS technology to silence *MbEGSs*. The transcription level of *MbEGSs* and *MbEOMT*s were measured using qRT-PCR in leaves inoculated with pNC-TRV2-*MbEGSs*. At 30 d after infection, the qRT-PCR results showed that the *MbEGS1* gene transcript level was decreased by 79.48% and the *MbEGS2* gene transcript level was increased 30.94 times in the pNC-TRV2-*MbEGS1* group in comparison to the pNC-TRV2-Control group. In the pNC-TRV2-*MbEGS2* group, the transcription level of *MbEGS2* decreased 90.35% and the transcription level of *MbEGS1* increased 3.11 times (Figure 4a). In addition, the transcription level of *MbEOMT1* and *MbEOMT2* also showed significant changes in different silencing groups (Figure 4b). The results showed that the transcription levels of *MbEOMT1* and *MbEOMT2* were increased 8.81 and 16.87 times in the *MbEGS1* silencing group. In the *MbEGS2* silencing group, the transcription levels of *MbEOMT1* and *MbEOMT2* were increased 6.56 and 8.69 times.

As a result of silencing *MbEGS1* and *MbEGS2*, the amount of methyleugenol in leaves significantly decreased compared to the pNC-TRV2-Control group, in the pNC-TRV2-*MbEGS1* group, the methyleugenol content decreased by 28.04%, and in the pNC-TRV2-*MbEGS2* group, it decreased by 19.45% (Figure 4c). In the VIGS treatment groups, no eugenol was detected in the total ion chromatogram of GC-MS (Supplemental Figure S1).

Collectively, these results indicated that *MbEGS1* and *MbEGS2* expression levels are correlated with methyleugenol content. The transcription levels of *MbEGS1* and *MbEGS2* directly affect the accumulation of methyleugenol in *M. bracteata*.

## 3. Discussion

Plant essential oils are aromatic, volatile liquids obtained from various plant parts and contain secondary metabolites from plants [36,37]. They are synthesized and stored in all plant organs, such as secretory cells, lumens, tubes, epidermal cells, and glandular hairs [38]. *M. bracteata* leaves contain significant amounts of essential oils, are acaricidal and attract fruit fly insects, and are a source of natural antibiotics and antioxidants [26,39]. *M. bracteata* essential oil has not only antibacterial and antioxidant properties but also apparent inhibitory effects on *Bacillus subtilis*, *Bacillus cereus*, *Phanerochaete chrysosporium,* and *Aspergillus flavus* [31]. In addition, the isolated methyleugenol, the principal active component of *M. bracteata* essential oil, exhibited a wide range of inhibition processes targeting the *C. violaceum* QS system [28]. Studies have shown that 29 components were identified from the essential oil of *M. bracteata* leaves, accounting for 96.49% of the total content, of which methyleugenol accounted for 90.46%. However, eugenol is not included in these 29 main components [27]. In addition, 25 main components were identified in the essential oil of *M. bracteata* leaves from different seasons in India, among which the relative content of methyleugenol was 87.2–89.5%, while eugenol was only detected in leaves in winter at 0.1% [29]. It can be seen that although *M. bracteata* leaves contain a large amount of methyleugenol, the content of eugenol is very low. After silencing *MbEGSs* separately, the content of eugenol in *M. bracteata* decreased, and the expression levels of *MbEOMT1* and *MbEOMT2* genes were significantly up-regulated, which may result in the complete conversion of eugenol to methyleugenol. Meanwhile, the content of eugenol could also be related to the tree age of *M. bracteata*. It may be for these reason that we could not identify eugenol accurately in the NIST 2011 mass spectrometry database.

According to previous studies, some EGS have very strict substrate specificity in substrate selection, while others can utilize different substrates. For example, ObEGS1 in *Ocimum basilicum* [10], CbEGS1 and CbEGS2 in *Clarkia breweri* [6], FaEGS1a and FaEGS1b in *Fragaria × ananassa* [40], *GoEGS* in *Gymnadenia odoratissima*, and *GcEGS* in *Gymnadenia conopsea* [41] can only catalyze the formation of a single eugenol from coniferyl acetate. Additionally, EGS in some plants can catalyze coniferyl acetate to produce eugenol and smaller isoeugenol, as demonstrated in the case of FaEGS2 in *Fragaria* × *ananassa* [40], GdEGS in *Gymnadenia densiflora* [41] and DcE(I) GS1 in *Daucus carota* subsp. *sativus* [42]. However, EGS of different species of *Ocimum*(*O. gratissimum* L.; *O. tenuiflorum* L. and *O. kilimandscharicum*) can catalyze coniferyl acetate and coumaryl acetate to generate eugenol and chavicol. When both substrates were used together, eugenol was predominantly detected while chavicol was present in trace amounts in the assays [13]. Thus, EGS in plants is primarily used to produce eugenol. In the analysis of the *M. bracteata* essential oil composition, methyleugenol was extremely high and isoeugenol was extremely low, while chavicol was not traced [27,29,30]. According to research, the transcription levels of EGS genes are directly related to eugenol content, whereas the transcription levels of EOMT genes are mainly related to methyleugenol content [43,44]. To verify whether the *MbEGS*s gene in *M. bracteata* can catalyze the formation of eugenol, during the initial stages of our research, we overexpressed the *M. bracteata MbEGS*s genes in *Fragaria vesca* fruits. We found that overexpression of *MbEGS1* and *MbEGS2* promoted the synthesis of eugenol [35]. It provides preliminary evidence that *MbEGS1* and *MbEGS2* can catalyze the synthesis of eugenol. However, we did not trace the concentrations of eugenol, isoeugenol, and chavicol in this study. Whether MbEGS catalyzes the formation of eugenol only by coniferyl acetate, which in turn produces methyleugenol remains to be further investigated.

EGS plays a key role in the production of eugenol and methyleugenol, but its regulation is unclear in *M. bracteata* methyleugenol biosynthesis. In order to further verify whether the *MbEGS1* and *MbEGS2* genes can regulate the biosynthesis of eugenol and methyleugenol in *M. bracteate,* we constructed overexpression vectors and VIGS vectors of *MbEGSs* genes and used agrobacterium-mediated and vacuum infiltration methods to infect *M. bracteata*. Based on the research results, the content of methyleugenol in the leaves of *M. bracteata* was significantly correlated with the transcription levels of *MbEGSs*. When the transcription levels of the *MbEGS1* and *MbEGS2* genes increased, the content of methyleugenol in the leaves of *M. bracteata* increased significantly. In contrast, silencing the *MbEGS1* and *MbEGS2* genes resulted in a significant decrease in the methyleugenol content. We also noted that silencing of *MbEGS*s resulted in a significant increase in the transcription of *MbEOMT1* and *MbEOMT2*. It may be that the silencing of *MbEGS* genes resulted in the reduction of eugenol content in *M. bracteata*. This triggers stress response in plants and causes them produce more methyleugenol by increasing transcription levels of the *MbEOMT* genes. However, even with increased *MbEOMT*s transcription levels, the content of methyleugenol in *M. bracteata* was still downregulated by 28.04% and 19.45% after silencing *MbEGS1* and *MbEGS2*. At the same time, *MbEGS1* gene silencing resulted in the up-regulation of *MbEGS2* gene transcription level by 30.94 times, while *MbEGS1* gene silencing resulted in the up-regulation of *MbEGS1* gene transcription level by 3.11 times. This indicated that the function of *MbEGS1* gene was stronger than that of *MbEGS2* gene, and *MbEGS*s are not redundant functions in *M. bracteata*. This study showed that the *MbEGS1* and *MbEGS2* genes were critical to the biosynthesis of methyleugenol in *M. bracteata* and were the key genes involved in this pathway. The GC-MS results of the overexpression and VIGS groups were compared with the NIST 2011 mass spectrometry database and showed that the matching degree of eugenol in the overexpression groups was less than 700, whereas it was not detected in the VIGS groups. In the biosynthesis of methyleugenol, eugenol is the only precursor. Yunxiang (*Rosa × hybrida*) flowers contained both eugenol and methyleugenol, and its relative eugenol content was higher than methyleugenol. While Yunxiang (*Rosa × hybrida*) flowers have a lower relative content of methyleugenol than *M. bracteata* leaves [45]. Thus, we speculate that both *MbEGS1* and *MbEGS2* are capable of catalyzing the conversion of coniferyl acetate into eugenol. However, due to the high transcription level of the *EOMT* gene in *M. bracteata*, eugenol is completely methylated by EOMT, producing a high quantity of methyleugenol. As a result, *M. bracteata* contains a relatively low amount of eugenol. Regarding the reasons for the efficient generation of methyleugenol in *M. bracteata*, it is necessary to investigate the in vitro enzymatic reactions, homology modeling, and site-directed mutagenesis of *MbEGS* and to conduct a functional investigation of the coupled reaction between MbEGS and MbEOMT.

## 4. Materials and Methods

### 4.1. Plant Materials

Transient expression technology can only be used to explore changes in gene expression levels for a short period of time. According to our pre-experiment results, the gene overexpression effect was the most effective at 48 h. Therefore, the materials used in the *MbEGSs* genes overexpression experiments were obtained from ten-year-old *M. bracteata* plants at Fujian Agriculture and Forestry University (Fuzhou, China). Test materials were the annual branches of *M. bracteata* with robust growth and a length of approximately 25 cm. Isolated branches were inserted into tissue culture flasks with clean water in a greenhouse with a 16 h light/8 h dark cycle at 25 °C for 5 d. A total of ten branches were taken from each treatment group and repeated three times. After 48 h of overexpression treatment, leaves were collected, snap-frozen in liquid nitrogen, and stored at −80 °C.

For the *MbEGSs* genes silencing experiments, our pre-experiment found that silence after 30 d of infection was most effective. Therefore, two-year-old *M bracteata* cutting seedlings with roots were selected for the VIGS experiments, and cutting seedling lengths were 20–25 cm. *M. bracteata* cutting seedlings were purchased from *Melaleuca bracteata* Nursery (Chengdu, China). Cutting seedlings were planted in the experimental greenhouse with a 16 h light/8 h dark cycle at 25 °C of the Institute of Horticultural Plants and Natural Products, Fujian Agriculture and Forestry University, and were used for infection experiments after 30 d of cultivation. A total of thirty cutting seedlings were taken from each treatment group and repeated three times. After VIGS infection, continue cultivation for 30 d, leaves were collected, quick-frozen in liquid nitrogen, and stored at −80 °C.

### 4.2. Total RNA Isolation and cDNA Synthesis

Total RNA was extracted from the *M. bracteata* leaves using the polysaccharide polyphenol plant Total RNA Extraction Kit (TianGen Biotech, Beijing, China) according to the manufacturer’s instructions. The concentration, purity (A260/280 = 1.8–2.1) and integrity of total RNA were measured using a Nano-400A Ultramicro nucleic acid analyzer (AllSheng Company, Hangzhou, China) and 1.0% agarose gel electrophoresis, respectively. First-strand cDNA was synthesized using One-Step gDNA Removal and cDNA Synthesis SuperMix Kit (TransGen Biotech, Beijing, China) following the kit’s instructions.

### 4.3. Target Fragment Amplification

Based on Iso-seq of the *M. bracteata* obtained by our laboratory, specific primers were designed using Primer Premier 5.0 software (Table 1). The synthetic first-strand cDNAs were diluted ten-fold for the first round of PCR amplification. The complete open reading frames (ORFs) and partial sequences of *MbEGS1* and *MbEGS2* were amplified by PCR Thermal Cycler (Bio-Rad company, Hercules, California, USA) according to 2×Taq Plus Master Mix II Kit (Vazyme, Nanking, China). The PCR reaction was performed in a 50 μL volume using the following conditions: pre-denaturation at 95 °C for 3 min, 35 thermal cycles (95 °C/15 s; 60 °C/20 s; 72 °C/60 s), and a final extension at 72 °C for 5 min. After the first round of PCR, the target fragment was recovered and purified using a TIANgel Purification Kit (TianGen Biotech, Beijing, China) after the PCR product was detected by 1% agarose gel electrophoresis. Next, primers containing Nimble cloning universal joints were used to conduct a second round of PCR, primers containing Nimble cloning universal joints are shown in Table 1. First-round PCR products were used as the templates, and PCR reaction procedure as above. After the second round of PCR amplification, the target amplified fragment containing Nimble cloning universal joints were obtained through a TIANgel Purification Kit (TianGen Biotech, Beijing, China).

### 4.4. Vector Construction

The overexpression vector containing the GUS reporter gene (pNC-Cam1304-MCS35S) and the VIGS expression vector (TRV1 and pNC-TRV2) were provided by the Chinese Academy of Tropical Agricultural Sciences (Haikou, China).

#### 4.4.1. Construction of Overexpression Vectors

The coding sequences of *MbEGS1* (GenBank: MW579500.1) and *MbEGS2* (GenBank: MW579501.1) have previously been deposited in the NCBI (National Center for Biotechnology Information, National Institutes of Health, Bethesda, Maryland, USA) database. To construct overexpression vectors pNC-Cam1304-MCS35S-*MbEGS1* and pNC-Cam1304-MCS35S-*MbEGS2*, the full length *MbEGS1* (963 bp) and *MbEGS2* (972 bp) open reading frames (ORFs) were cloned from *M. bracteata*. The full length primers containing Nimble cloning universal joints were amplified by secondary PCR, and the full length products containing the universal joint were purified. The purified products were cloned into the pNC-Cam1304-MCS35S vector using the Nimble Cloning kit (Yi Tian Biotech, Haikou, China) following the kit’s instructions. Overexpression vector carrying the full length were transformed into 50 μL *E. coli* T1 cells using the T1 Cloning Kit (TransGen Biotech, Beijing, China) and selected to be cultured on LB (Luria Bertani Medium) plates containing 100 mg·L^−1^ kanamycin at 37 °C overnight. As a next step, the six monoclonal colonies were selected and cultured for 12 h at 37 °C in 700 μL LB liquid medium containing kanamycin. Through PCR verification, 300 μL of the positive clones bacteria solution was selected and sent to BioSune (BioSune Co., Ltd., Shanghai, China) for sequencing. The remaining positive clones bacteria solution were added with 60% glycerin at a ratio of 1:1 and stored at −80 °C.

The sequencing results demonstrated that the full length were successfully linked to the overexpression vector. About 100 μL positive clones bacteria solution were cultured in 5 mL LB liquid medium containing kanamycin at 200 rpm in a shaker for 16 h at 37 °C. Recombinant plasmids were extracted from bacterial solution using the TIANprep Mini Plasmid Kit (TianGen Biotech, Beijing, China) according to the manufacturer’s instructions. The concentration and integrity of plasmids were measured using a Nano-400A Ultramicro nucleic acid analyzer (AllSheng Company, Hangzhou, China) and 1.0% agarose gel electrophoresis, respectively. Finally, recombinant vector plasmids were introduced into *Agrobacterium tumefaciens* strain GV3101 (Zoman Biotechnology Co., Ltd., Beijing, China) using the freeze-thaw method and selected to be cultured on YEB (Agrobacterium rhizogene Medium) plates containing 100 mg·L^−1^ kanamycin and 50 mg·L^−1^ rifampicin. Positive clones were verified and stored as described above. *A. tumefaciens* strain GV3101 carrying the pNC-Cam1304-MCS35S-Control, pNC-Cam1304-MCS35S-*MbEGS1*, pNC-Cam1304-MCS35S-*MbEGS2* vectors were constructed.

#### 4.4.2. Construction of VIGS Vectors

To construct VIGS vectors pNC-TRV2-*MbEGS1* and pNC-TRV2-*MbEGS2,* 298 bp *MbEGS1* fragment and 241 bp *MbEGS2* fragment (Supplemental Figure S2) were selected and cloned into the pNC-TRV2 vector with Nimble Cloning kit (Yi Tian Biotech, Haikou, China). The TRV1 and pNC-TRV2 combined plasmids (pNC-TRV2-Control, pNC-TRV2-*MbEGS1* and pNC-TRV2-*MbEGS2*) were inoculated to *E. coli* T1 cells and *A. tumefaciens* strain GV3101 using the method as above. Finally, *A. tumefaciens* strain GV3101 carrying the TRV1, pNC-TRV2-Control, pNC-TRV2-*MbEGS1,* and pNC-TRV2-*MbEGS2* vectors were constructed and stored at −80 °C.

### 4.5. Infection Preparation and Vacuum Infiltration

For the initial activation, 50 μL of *A. tumefaciens* strains GV3101 containing the recombinant vectors (pNC-Cam1304-MCS35S-Control, pNC-Cam1304-MCS35S-*MbEGS1*, pNC-Cam1304-MCS35S-*MbEGS2,* TRV1, pNC-TRV2-Control, pNC-TRV2-*MbEGS1,* and pNC-TRV2-*MbEGS2*) were taken, placed in 5 mL of YEB medium containing 100 mg·L^−1^ kanamycin and 50 mg·L^−1^rifampicin, and cultured at 28 °C for 12 h in a 200 rpm shaker. Afterward, 1 mL of primary activated propagation solution was added to 100 mL of YEB liquid medium (containing Kan and Rif), placed in a 200 rpm shaker and incubated at 28 °C for 16 h in the dark until the OD_600_ value reached approximately 1.0. Agrobacterium cells containing the different constructs were harvested at 12,000 rpm for 10 min and resuspended in Agrobacterium infiltration buffer (10 mM MES pH 5.6–5.7, 10 mM MgCl_2_, 0.1 mM AS), and the final OD_600_ value was adjusted to 1.0.

Additionally, in the VIGS experiment, *Agrobacterium* suspension containing the pNC-TRV2-Control, pNC-TRV2-*MbEGS1* and pNC-TRV2-*MbEGS2* vectors were mixed with *Agrobacterium* suspension TRV1 at a volume ratio of 1:1. The mixed bacterial fluids were named VIGS-TRV2, VIGS-MbEGS1, and VIGS-MbEGS2, and the infection fluids needed for overexpression and VIGS were placed in the dark for 3 h.

*Agrobacterium* infection solutions from different treatment groups were fully contacted with plant materials, and subjected to vacuum (−70 kPa) for 20 min. After infection, branches treated with overexpression were cultured in clean water for 48 h, and then the leaves were collected in groups. However, the VIGS-treated cutting seedlings were planted in the artificial climate room of the College of Horticulture, Fujian Agriculture and Forestry University, and leaves were collected after a month.

### 4.6. Detection of the GUS Reporter Gene and TRV Virus

To verify whether the plant materials were successfully infected, total RNA was extracted from different treatment groups and reverse transcribed into cDNA. The primers for the *GUS* and *TRV2* genes are described in Table 1. We tested the *GUS* and *TRV2* genes by PCR.

### 4.7. Quantitative RT-PCR Analysis

Total RNA extraction and cDNA synthesis were performed as described above. A five-fold dilution of the cDNA was used for qRT-PCR. qRT-PCR determination was performed using a TB Green^®^ *Premix Ex Taq*^™^ kit with a total reaction volume of 20 μL (Takara Biotech, Beijing, China). The gene-specific primers used for qRT-PCR were designed using Primer Premier 5.0 software. qRT-PCR specific primers were qMbEGS1, qMbEGS2, qMbEOMT1, qMbEOMT2, and qMbCYP, and the specific sequences are shown in Table 1. In this study, qRT-PCR assays were performed using a LightCycler 96 (LightCycler^®^ 96, Roche Company, Basel, Switzerland), and the qRT-PCR conditions were as follows: predenaturation at 95 °C for 30 s; 40 cycles of denaturation for 5 s at 95 °C and annealing for 30 s at 60 °C; and a final signal acquisition at 72 °C for 30 s. The 2^−ΔΔCT^ method was used to analyze the qRT-PCR expression data according to Gong et al. [46]. The data were analyzed by variance using SPSS software.

### 4.8. Determination of Eugenol and Methyleugenol Content

*M. bracteata* leaves from different treatment groups were ground in liquid nitrogen into powder. For each treatment group, 0.20 g leaf powder was weighed separately, and extraction with 5 mL petroleum ether was conducted in a shaker at 200 rpm for 3 h at 28 °C. Following centrifugation at 12,000 rpm for 10 min, the supernatants were extracted using sterile syringes of 2 mL and each extract was passed through a membrane filter (0.22 mm). For each sample, three biological replicates were conducted. The eugenol and methyleugenol contents were detected by GC680 + SQ8T + HS40 gas chromatography-mass spectrometry (GC-MS, Perkin Elmer Company, Waltham, Massachusetts, USA). The GC-MS setup parameters were according to Li et al. [30]. GC-MS detection results were compared with the mass spectrometry database of NIST 2011, if the matching degree is greater than 750 (the total value is 1000), and the probability of the first matching compound is as higher as 100 than the second possible compound, then we defined the first matching compound as complete identification; if the matching degree is greater than 700, but not confirmed by the previous literature, then we defined the result as preliminary identification; if the matching degree is less than 700, the compound has a low level of content that cannot be accurately identified.

Finally, the contents of eugenol and methyleugenol were calculated by area normalization and external standard method. The eugenol and methyleugenol standards (Solarbio Science and Technology Co., Ltd., Beijing, China) were formulated into different concentration gradients (6.25; 12.50; 25.00; 50.00; 100.00; 200.00 μg/mL) using petroleum ether. Eugenol and methyleugenol peak areas were determined in 2 mL solutions under the same GC-MS conditions. The linear regression equation was obtained by taking the concentration of eugenol and methyleugenol as the horizontal coordinate and the absorption peak area as the vertical coordinate.

## 5. Conclusions

In this study, we found that the content of methyleugenol in the leaves of *M. bracteata* was significantly correlated with the transcription levels of *MbEGS1* and *MbEGS2* genes. We suggest that MbEGS1 and MbEGS2 are involved in methyleugenol biosynthesis in *M. bracteata* and are the key genes involved in this pathway.

## Figures and Tables

**Table 1:** List of primers used in this study.

Primer Name	Forward Primer Sequence (5′→3′)	Reverse Primer Sequence (5′→3′)
MbEGS1	ATGGCAGGAGAGGCCGAGAAAA	TCATTCGAAAGCAGCCCTAGCTG
MbEGS2	ATGACGATCATAAGCAGTAGTTGCA	TCATTCCAATACAGCGCTTGC
NC-MbEGS1	agtggtctctgtccagtcctATGGCAGGAGAGGCCGAGAAAA	ggtctcagcagaccacaagtTCATTCGAAAGCAGCCCTAGCTG
NC-MbEGS2	agtggtctctgtccagtcctATGACGATCATAAGCAGTAGTTGCA	ggtctcagcagaccacaagtTCATTCCAA TACAGCGCTTGC
VIGS-MbEGS1	GCAGGCATTCCTTACACC	CGGGCACTTGGACTTTC
VIGS-MbEGS2	GCACTCCCACCATTCG	AGTCGTTCGCCACTTTT
NC-VIGS-MbEGS1	agtggtctctgtccagtcctGCAGGCATTCCTTACACC	ggtctcagcagaccacaagtCGGGCACTTGGACTTTC
NC-VIGS-MbEGS2	agtggtctctgtccagtcctGCACTCCCACCATTCG	ggtctcagcagaccacaagtAGTCGTTCGCCACTTTT
GUS	ACGGGGAAACTCAGCAAGC	ATGTAATGTTCTGCGACGCTCA
TRV2	GGCGGTTCTTGTGTGTCAAC	GGCGGTTCTTGTGTGTCAAC
qMbEGS1	TTCGGGAGTGAGGAGGATAA	TGGGGATGAAGGAGGTTGTT
qMbEGS2	CCCCACCTTTGTCTTCGCTC	AACCTCTTTATGTTGCCCGC
qMbEOMT1	CACCAACATACGACGGAATCACTCA	TCATCGAACATGCCATCGCCTTC
qMbEOMT2	CCTTGCTCCGATGGTGCTGATG	ATGCGATGCCACCGTTCTTGAC
qMbCYP	CGCCGTGAAGGGATGTTTGT	ACGGGAAGCATTTCATCCTCTG

Note: Underlined bases indicate Nimble cloning universal joints.

## Data Availability

Not applicable.

## Supplementary Materials

The following supporting information can be downloaded at: https://www.mdpi.com/article/10.3390/plants12051026/s1. *Melaleuca bracteata* eugenol synthase 1 (EGS1) mRNA, complete cds (https://www.ncbi.nlm.nih.gov/nuccore/MW579500.1 (accessed on 13 December 2022), GenBank: MW579500.1); *Melaleuca bracteata* eugenol synthase 2 (EGS2) mRNA, complete cds (https://www.ncbi.nlm.nih.gov/nuccore/mw579501 (accessed on 13 December 2022), GenBank: MW579501.1). Figure S1. GC-MS analysis of VIGS experiments. Figure S2. Cloning of *MbEGS1*, *MbEGS2* gene fragments (A), and cloning of *MbEGS1*, *MbEGS2* gene fragments with NC linkers (B).
