# Supplementary material for: The Role of MbEGS1 and MbEGS2 in Methyleugenol Biosynthesis by Melaleuca bracteata
Source: Plants (Basel). 2023 Feb 24;12(5):1026. doi: 10.3390/plants12051026 (PMC10005710; doi:10.3390/plants12051026)
Supplement: Supplementary file 1 [file plants-12-01026-s001.zip › plants-2179054-supplementary.pdf]

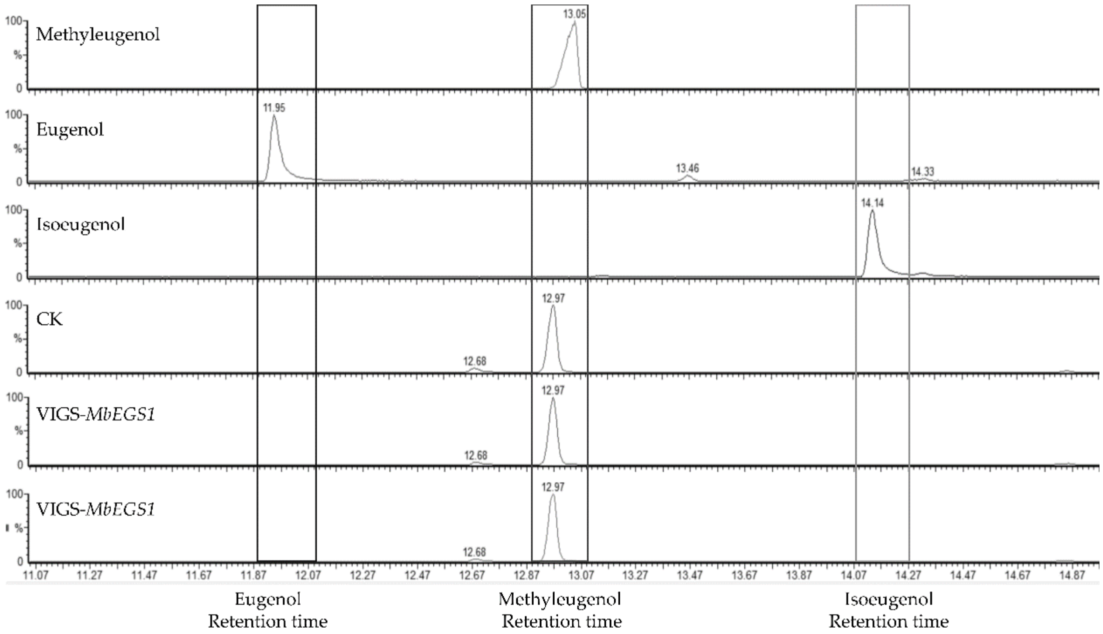

Figure S1. GC-MS analysis of VIGS experiments

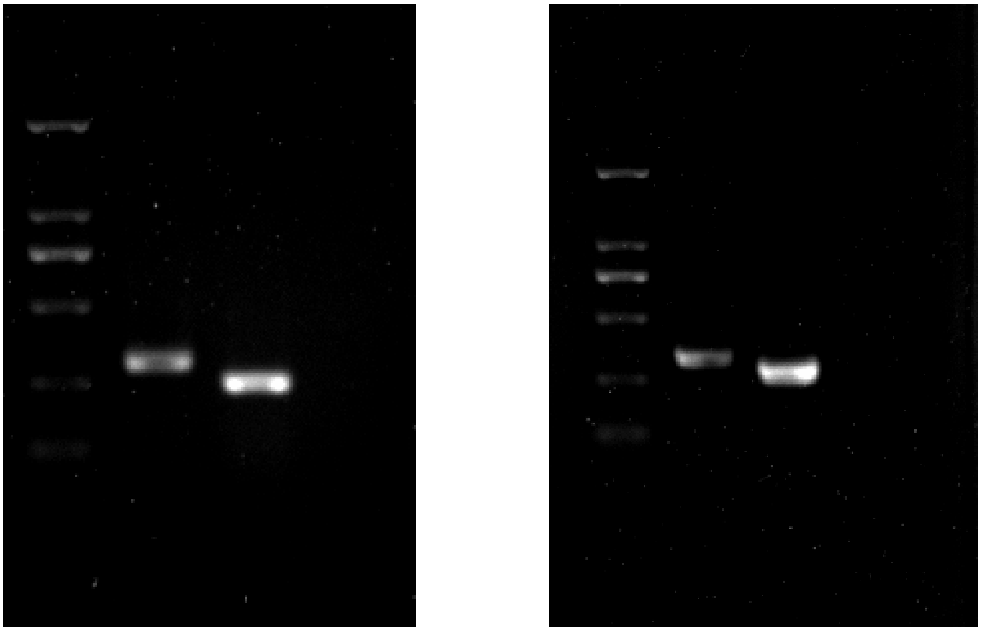

(**a**) (**b**)

Figure S2. Cloning of *MbEGS1*, *MbEGS2* gene fragments (A), and cloning of *MbEGS1*, *MbEGS2* gene fragments with NC linkers (B) in VIGS experiments
